# Displacement Transmissibility Analysis of Stewart Platform Based SINS’s Bumper Under Base Vibration Excitation

**DOI:** 10.3390/s25113434

**Published:** 2025-05-29

**Authors:** Yongqiang Tu, Haoran Zhang, Hao Wu, Yintao Li, Baohua Bao, Gang Lu, Hongwei Lin, Xinkai Chen, Jianyu Fan

**Affiliations:** College of Marine Equipment and Mechanical Engineering, Jimei University, Xiamen 361021, China; 202361000149@jmu.edu.cn (Y.T.); 202412855070@jmu.edu.cn (H.Z.); 202321361131@jmu.edu.cn (H.W.); lyt200207@163.com (Y.L.); 202321365108@jmu.edu.cn (B.B.); 202321367045@jmu.edu.cn (G.L.); 202321361061@jmu.edu.cn (H.L.); 202321361104@jmu.edu.cn (X.C.)

**Keywords:** displacement transmissibility, vibration isolation, Stewart platform, bumper, strap-down inertial navigation system

## Abstract

The Stewart platform based bumper is essential for the strap-down inertial navigation system (SINS) to attenuate the vibration excitation from base to SINS. Displacement transmissibility is the most important performance indicator to quantify the vibration isolation effectiveness of the bumper. In this paper, considering the structural complexity and dynamic coupling of the bumper in parallel mechanism shape, a novel method of displacement transmissibility analysis for the bumper under base vibration excitation is proposed. Firstly, a lumped parameter model is established for the bumper by defining dynamic matrices, which includes stiffness matrix, damping matrix and mass matrix. Secondly, coupled dynamic equations of the bumper under base vibration excitation are derived based on the proposed model, and the coupled dynamic equations are transferred to decoupled dynamic equations by decoupling method. Thirdly, a calculation flowchart of the vibration isolation performance for the bumper is proposed based on the deduced decoupled dynamic equations, and theoretical results for displacement transmissibility are obtained by the calculation flowchart. Finally, the proposed analysis approach of displacement transmissibility for the bumper is validated by vibration experiments as the maximum quantitative gap between theoretical and experimental results is 3.6%.

## 1. Introduction

The strap-down inertial navigation system (SINS), as a precision navigation device extensively employed in marine applications, is rigidly mounted on vessel platforms to deliver real-time positioning coordinates and three-dimensional attitude parameters with high accuracy [1,2,3]. In practice, the ship-borne SINS is always directly affected by interferences from the surrounding environment like huge impact and vibration due to explosions and sea waves which will degrade the precision of the SINS rapidly and even cause the failure of system functions and the destruction of structures [4]. To address this issue, a bumper must be integrated into the structural interface between the mounting base and the SINS, effectively dampening high-amplitude impact loads and broadband vibrational energy transmitted from marine vessel platforms to the SINS unit during marine operations.

Furthermore, the SINS vibration isolation system must achieve dual functionality: vibration attenuation and kinematic retraction precision (defined as angular displacement deviations between the dynamic vessel structure and inertial reference frame during vibration transients), given the system’s critical requirement for maintaining angular reference stability at sub-milliradian levels during vessel maneuvering. Owing to their superior performance in dynamic bandwidth, structural load capacity, micron-level positioning resolution, optimized stiffness-to-weight ratio and thermal stability, Stewart platform based bumpers have been widely used in vibration isolation for precision instruments [5,6,7]. Considering the restoration accuracy requirement for the SINS’s bumper, paper [8] designed a bumper based on a Stewart platform to reduce the influence of huge impact and vibration on the performance of the SINS from base. However, paper [8] only focused on the geometric configuration optimal design for restoration accuracy of the bumper. However, vibration isolation performance of the bumper has not been studied, but vibration isolation performance is a vital issue in evaluating the bumper and optimizing the buffer bars. Especially, transmissibility is the most important indicator to quantify the effectiveness of vibration isolation systems [9,10,11,12,13,14]. In order to evaluate the vibration isolation performance of the bumper, transmissibility analysis needs to be studied.

More recently, studies of transmissibility analysis for vibration isolators have been focused on by plenty of scholars. Carrella et al. [15] derived an expression of the force transmissibility for quasi-zero-stiffness (QZS) isolator using the harmonic balance method (HBM). Expressions of force and displacement transmissibility for a nonlinear isolator with high-static-low-dynamic-stiffness (HSLDS) elements were analyzed in paper [16]. Paper [17] obtained the displacement transmissibility for nonlinear vibration isolators using the HBM and the Runge–Kutta method. Lu et al. [18] studied transmissibility using the root-mean-square (RMS) ration as the response to the excitation for a nonlinear vibration isolation system. Shi et al. [19] investigated vibration transmission characteristics for oscillators.

All transmissibility analyses for vibration isolators mentioned previously derived expressions of transmissibility by modeling the dynamic system as a single DOF system. However, a Stewart platform based a SINS’s bumper is a complicated parallel structure which has six DOF [20,21,22], and there are significant differences between a single DOF system and a six DOF system in dynamic modeling and transmissibility analysis due to dynamic coupling between different directions [23,24,25]. A transmissibility analysis of Stewart platform based isolators should be separately investigated from studies of single DOF isolation systems. Zhou et al. [26] estimated the effectiveness of transmissibility for a 6-DOF platform. Zheng et al. [27] obtained both the force and displacement transmissibility by expressing dynamic equation in Laplace domain. Paper [28] analyzed the anti-vibration performance of a 6-DOF passive vibration isolator containing X-shape supporting structures by studying the displacement transmissibility.

All these studies for transmissibility of Stewart platform based isolators above proposed a simple and quick evaluation method simplifying the geometry complexity of the parallel isolator by ignoring the dynamic coupling of the platform. However, a Stewart platform based SINS’s bumper is a complicated parallel structure whose dynamic coupling is common and non-negligible [29], and the dynamic coupling has an impact on the dynamics of mechanical systems [30,31] as well as the transmissibility of the bumper, so a full research study of transmissibility for the Stewart platform based SINS’s bumper considering the dynamic coupling should be performed.

The transmissibility is divided into two types: one is displacement transmissibility to describe the damping capacity to protect equipment on the bumper from vibration excitation from base while the equipment on the bumper do not produce force disturbance; the other is force transmissibility to describe the damping capacity to protect equipment in the ground from the force disturbance of the equipment fixed on the bumper, as equipment on the bumper is an interference source. Because the SINS does not produce force, and the purpose of Stewart platform based bumper is to attenuate the vibration excitation from base to SINS, we only focus on the displacement transmissibility for the bumper instead of force transmissibility.

In this paper, for the purpose of full study of displacement transmissibility for the Stewart platform based SINS’s bumper, a novel approach for displacement transmissibility analysis of the bumper under base vibration excitation is proposed considering the dynamic coupling of the 6-DOF Stewart system. The contributions of the article are as follows.

(1) A dynamic model for the bumper is established.
(2) Dynamic equations of the bumper under base vibration excitation are derived.
(3) A calculation flowchart of the vibration isolation performance of the bumper is proposed.

The remainder of this paper is organized as follows. Section 2 gives an overview of the Stewart platform based SINS’s bumper. In Section 3, a lumped parameter model for the bumper is established. In Section 4, a calculation flowchart of the vibration isolation performance of the bumper is proposed. In Section 5, a vibration experiment is conducted to verify the proposed method. Finally, conclusions are summarized in Section 6.

## 2. Stewart Platform Based SINS’s Bumper

### 2.1. Mechanical Configuration

**Figure 1:**
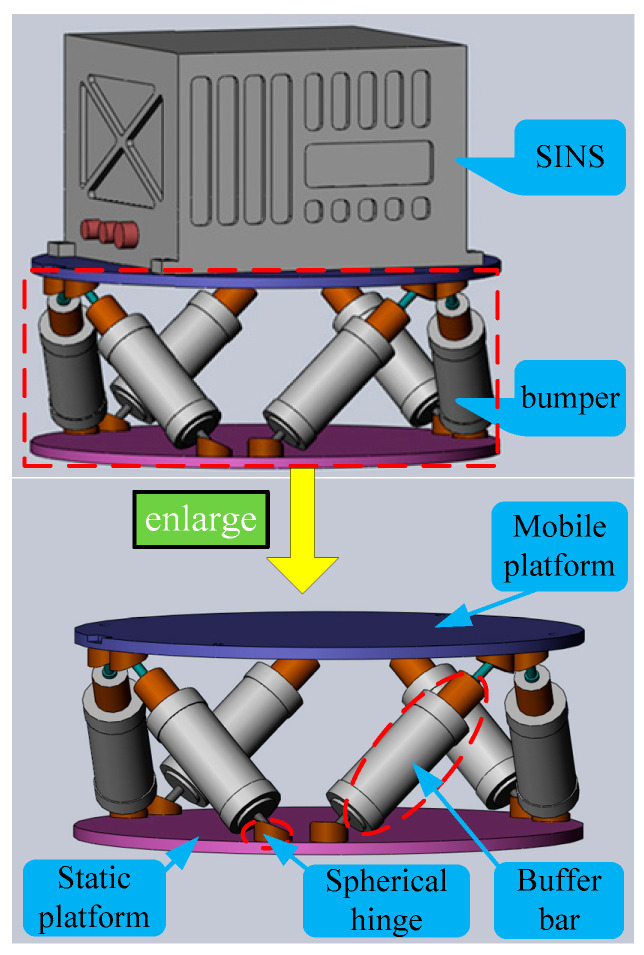
Mechanism composition diagram of Stewart platform based SINS’s bumper.

The bumper as shown in Figure 1 is mounted on the mounting base of moving ships, and the navigation system is securely mounted on the vibration isolation device’s moving platform. The bumper consists of a mobile platform, a static platform, six buffer bars of uniform length and twelve uniformly distributed spherical hinges. Its symmetrical configuration stems from the identical length of all buffer bars and the uniform distribution of spherical hinges. This symmetrical Stewart platform, characterized by six linear elements and twelve spherical hinges, is formally classified as a 6-SPS (Spherical-Prismatic-Spherical) parallel mechanism [32]. The 6-SPS parallel mechanism, possessing six DOF [33], enables the bumper to isolate vibrations in all six directions of the mounting base.

**Figure 2:**
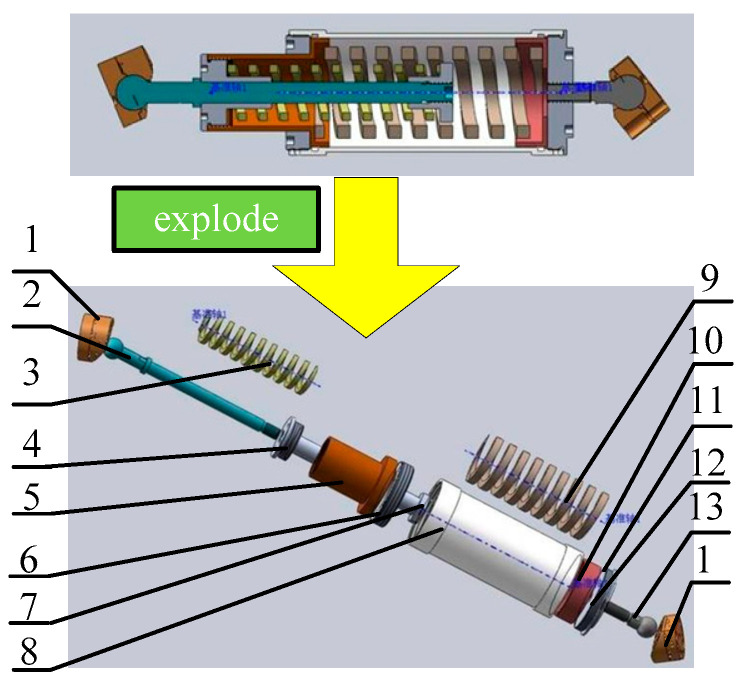
Cutaway view and explosive view of buffer bar (1—spherical-hinge base; 2—upper ball rod; 3—upper spring; 4—upper limit cap; 5—upper cylinder; 6—upper cover; 7—adjustment nut; 8—lower cylinder; 9—lower spring; 10—cover; 11—lower limit cap; 12—adjusting shim; 13—lower ball rod).

The vibration isolation mechanism is illustrated in Figure 2 through a cutaway assembly and exploded view of the buffer bar. This component incorporates dual helical compression springs with identical stiffness and damping parameters. Constrained by cylindrical housings, the springs undergo asynchronous compressive motion, resulting in a bidirectional linear buffer mechanism with equivalent dynamic characteristics in both loading directions. During base excitation, the buffer bars convert kinetic energy through axial displacement, progressively transferring vibration energy to spring potential energy before ultimately dissipating it as internal thermal energy.

### 2.2. Geometric Configuration

**Figure 3:**
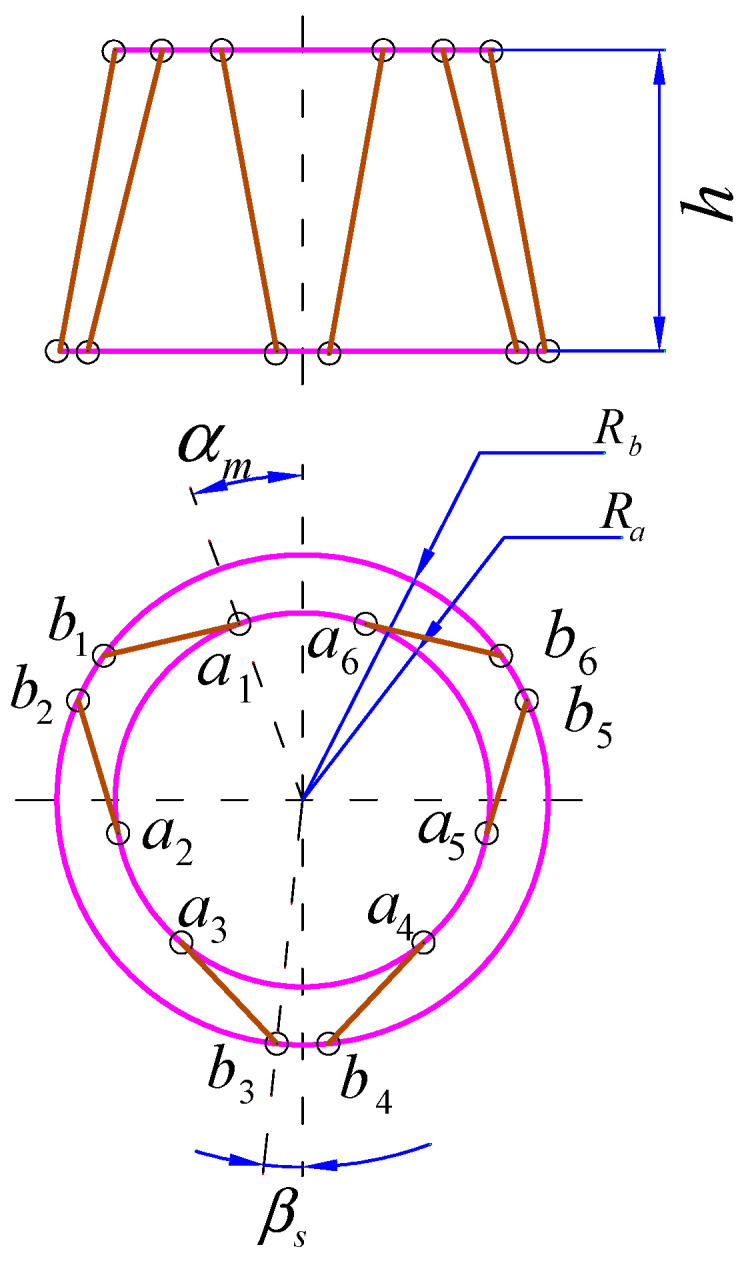
Geometric configuration of the bumper.

As shown in Figure 3, geometric configuration of the bumper depends on structural parameters including height h; the radius of the static platform $R_{b}$; the radius of the mobile platform $R_{a}$; the half flare angle of adjoining spherical hinge on static platform $\beta_{s}$ and the half flare angle of adjoining spherical hinge on mobile platform $\alpha_{m}$, and all structural parameters of the bumper are listed in Table 1.

## 3. Dynamic Model and Equations

In order to analyze the displacement transmissibility of the bumper, dynamic equations under base vibration excitation should be established. Firstly, complete dynamic equations considering the dynamic coupling of the bumper are set up. Then the coupled dynamic equations are transferred to decoupled dynamic equations by decoupling method. It should be noticed that some simplifications are made during the equations’ derivation process according to the practice bumper as follows.

(1) Geometry variation of the bumper during vibration can be neglected because the base vibration excitations are small, so Jacobian matrix is regarded as a constant matrix dependent on the static configuration of the bumper, and dynamic matrices of the bumper are also constant matrices because Jacobian matrix is a constant matrix.
(2) The force on the buffer bars is mainly completed by the spring inside the buffer bars, and the mass of the outer shell of the buffer bar itself is relatively small compared to the load weight, so the kinetic energy of buffer bars is neglected.
(3) All joints are regarded as ideal spherical, so their clearance and stiffness are neglected.

### 3.1. Coupled Dynamic Equations

**Figure 4:**
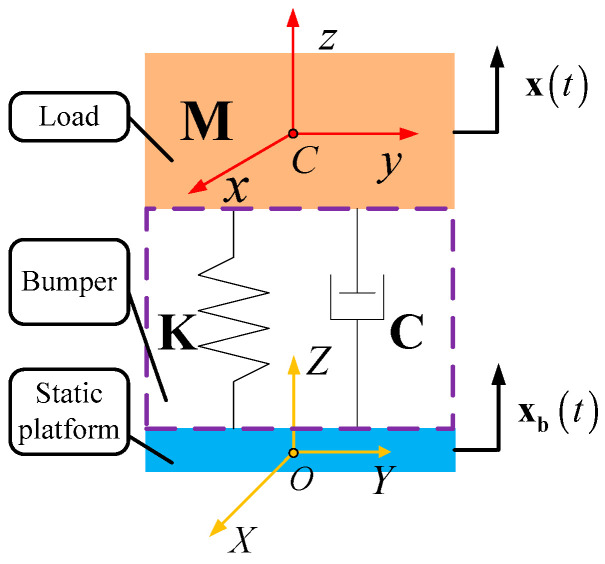
Lumped parameter model for system of bumper and SINS under base vibration excitation.

The system of Stewart platform based bumper and SINS is regarded as a lumped parameter model illustrated in Figure 4.

In Figure 4, point O is the center of mass of the static platform, and the static platform frame O−XYZ is fixed on the static platform. As the weight of buffer bars is negligible related to the weight of the SINS and mobile platform, the weight of buffer bars is ignored, and the load of bumper is regarded as the assembly of the SINS and mobile platform. Point C is the center of mass of the load, and the load frame C−xyz is bound on the load. Vector x is set as the generalized coordinate of the mobile platform in O−XYZ and can be written as follows.

(1) $$x={\left[\begin{array}{cc} p & \Phi \end{array}\right]}^{T}={\left[x_{p},y_{p},z_{p},\alpha ,\beta ,\gamma \right]}^{T},$$

where vector $p={\left[\begin{array}{ccc} x_{p} & y_{p} & z_{p} \end{array}\right]}^{T}$ is defined as the position vector of point C in O−XYZ, and Euler angles $\Phi ={\left[\begin{array}{ccc} \alpha & \beta & \gamma \end{array}\right]}^{T}$ is defined as the attitude of mobile platform related to static platform, in which mobile platform rotates α, β and γ around axis OX, OY and OZ in sequence.

The base vibration excitation is transferred to static platform directly, so the movement of static platform represents the base vibration excitation. Vector $x_{b}$ is set as the generalized coordinate of the static platform in static O−XYZ (the static O−XYZ is a static frame which coincides with the initial O−XYZ when the static platform is in static) as well as the base vibration excitation, and it can be written as follows.

(2) $$x_{b}={\left[\begin{array}{cc} p_{b} & \Phi_{b} \end{array}\right]}^{T}={\left[x_{pb},y_{pb},z_{pb},\alpha_{b},\beta_{b},\gamma_{b}\right]}^{T},$$

where vector $p_{b}={\left[\begin{array}{ccc} x_{pb} & y_{pb} & z_{pb} \end{array}\right]}^{T}$ is defined as the position vector of point O in static O−XYZ, and Euler angles $\Phi_{b}={\left[\begin{array}{ccc} \alpha_{b} & \beta_{b} & \gamma_{b} \end{array}\right]}^{T}$ is defined as the attitude of static platform related to static O−XYZ, in which static platform rotates $\alpha_{b}$, $\beta_{b}$ and $\gamma_{b}$ around static axis OX, OY and OZ of static O−XYZ in sequence.

The dynamic characteristic of the lumped parameter model depends on three dynamic matrices of the bumper: mass matrix M; stiffness matrix K and damping matrix C, and the expressions of the three dynamic matrices and the detailed derivation process are provided in Appendix A.

According to Figure 4 and the definition of kinematic variables as well as dynamic matrices of the bumper, the dynamic equations of the bumper under base vibration excitation can be written in matrix form as follows.

(3) $$M\ddot{x}+C\left(\dot{x}-{\dot{x}}_{b}\right)+K\left(x-x_{b}\right)=0,$$

Calculations of dynamic matrices M, K and C in Equation (3) using expressions given in Appendix A indicate that all these matrices are non-diagonal matrices, so Equation (3) is a set of coupling equations which cannot be solved analytically.

In order to analyze transmissibility of the bumper analytically, Equation (3) is transferred to a decoupling equation set using decoupling method.

### 3.2. Decoupling Method

Coupled dynamic Equation (3) can be written in variables-separated form as follows.

(4) $$M\ddot{x}+C\left(\dot{x}-{\dot{x}}_{b}\right)+K\left(x-x_{b}\right)=0,$$

According to the Equations (A15) and (A16) for K and C in Appendix A, the relationship of K and C can be written as follows.

(5) C=λK,

where λ is the ratio of damping to stiffness for buffer bars defined as λ=c/k, and k is the stiffness of buffer bar, and c is the damping of buffer bar.

Because C=λK, Equation (4) can be decoupled.

Firstly, intermediate variables q and $q_{b}$ are applied to replace the vectors x and $x_{b}$, which are defined by coordinate transformation for x and $x_{b}$ shown as follows.

(6) $$\begin{array}{l} x=M^{-\frac{1}{2}}q\\ x_{b}=M^{-\frac{1}{2}}q_{b} \end{array},$$

Equation (6) is substituted into Equation (4), and then both sides of Equation (4) are left multiplied by $M^{-\frac{1}{2}}$; then Equation (4) can be written as follows.

(7) $$\ddot{q}+{Κ}_{M}q+C_{M}\dot{q}={Κ}_{M}q_{b}+C_{M}{\dot{q}}_{b},$$

where ${Κ}_{M}=M^{-\frac{1}{2}}KM^{-\frac{1}{2}}$, $C_{M}=M^{-\frac{1}{2}}CM^{-\frac{1}{2}}$.

Then, ${Κ}_{M}$ and $C_{M}$ are diagonalized by eigenvalue decomposition. Then regularized eigenvectors of ${Κ}_{M}$ constitute a matrix as follows.

(8) $$P=\left[\begin{array}{cccccc} \nu_{1} & \nu_{2} & \nu_{3} & \nu_{4} & \nu_{5} & \nu_{6} \end{array}\right],$$

where $\nu_{i}\left(i=1,2,\ldots ,6\right)$ are regularized eigenvectors of ${Κ}_{M}$.

Coordinate transformation for q and $q_{b}$ are shown as follows.

(9) $$\begin{array}{l} q=Pu\\ q_{b}=Pu_{b} \end{array},$$

Equation (9) is substituted into Equation (7), and then both sides of Equation (7) are left multiplied by $P^{T}$. Because the eigenvector matrix P is an orthogonal matrix, the $P^{T}$ multiplied by P is equal to the identity matrix, so Equation (7) can be written as follows.

(10) $$\ddot{u}+{Κ}_{d}u+C_{d}\dot{u}={Κ}_{d}u_{d}+C_{d}{\dot{u}}_{d},$$

where u, $\dot{u}$ and $\ddot{u}$ are generalized displacement, velocity and acceleration of the load in modal coordinate, and $u_{b}$, ${\dot{u}}_{b}$ are base vibration displacement and velocity vector in modal coordinate. According to the deduction process of Equation (10), ${Κ}_{d}=P^{T}{Κ}_{M}P$, $C_{d}=P^{T}C_{M}P$. Meanwhile, ${Κ}_{d}$ and $C_{d}$ are both diagonal matrices as presented in Equations (11) and (12).

(11) Κd=ω12 0 ⋱ 0 ω626×6,

(12) Cd=2ξ1ω1 0 ⋱ 0 2ξ6ω66×6,

where $\omega_{i}\left(i=1,2,\ldots ,6\right)$ is modal frequency, and $\xi_{i}\left(i=1,2,\ldots ,6\right)$ is modal damping ratio.

According to Equations (6) and (9), the coordinate transformation between actual physical coordinate x and modal coordinate u is shown as follows.

(13) $$\begin{array}{l} x=Su;u=S^{-1}x\\ x_{b}=Su_{b};u_{b}=S^{-1}x_{b} \end{array},$$

where S is the transformation matrix, and it can be written as follows.

(14) $$S=M^{-\frac{1}{2}}P,$$

Decoupled solutions in modal coordinate can be transferred to solutions in physical coordinate using Equation (13).

### 3.3. Decoupled Dynamic Equations

According to Equations (11) and (12), coupled Equation (10) in physical coordinate can be written as six ordinary differential equations which are independent of each other in modal coordinate as follows.

(15) $${\ddot{u}}_{i}\left(t\right)+2\xi_{i}\omega_{i}{\dot{u}}_{i}\left(t\right)+\omega_{i}^{2}u_{i}\left(t\right)=\omega_{i}^{2}u_{bi}\left(t\right)+2\xi_{i}\omega_{i}{\dot{u}}_{bi}\left(t\right),\left(i=1,2,\ldots ,6\right),$$

where $u_{i}\left(t\right)$ and $u_{bi}\left(t\right)$ are the vibration response of the load and base vibration excitation for i order modal shape in modal coordinate, respectively.

## 4. Displacement Transmissibility of the Bumper

### 4.1. Calculation Flowchart for Response of the Load

**Figure 5:**
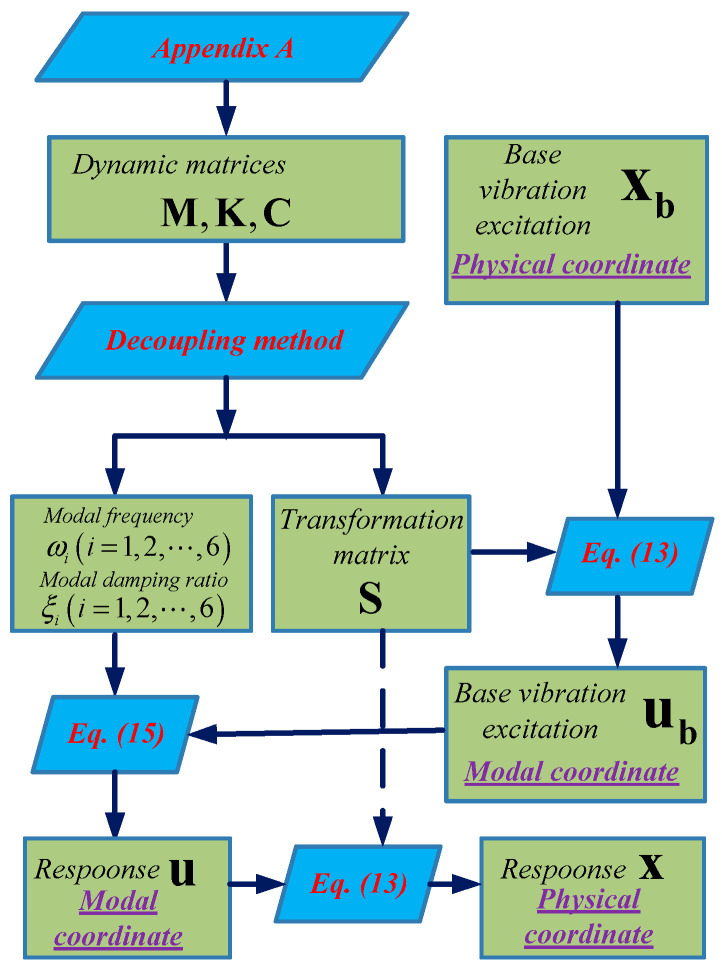
Calculation flowchart for response of the load under base vibration excitation.

The purpose of vibration isolation performance analysis of the bumper is to obtain the response of the load in physical coordinate when base vibration excitation is known. The calculation flowchart for response of the load under base vibration excitation is presented in Figure 5. Dynamic matrices M, K and C are calculated based on the equations in Appendix A firstly. Then modal frequency $\omega_{i}\left(i=1,2,\ldots ,6\right)$, modal damping ratio $\xi_{i}\left(i=1,2,\ldots ,6\right)$ and transformation matrix S are obtained using the decoupling method presented in Section 3. Next, the known base vibration excitation $x_{b}$ in physical coordinate and the matrix S are substituted into Equation (13) to obtain base vibration excitation $u_{b}$ in modal coordinate. Afterwards, $u_{b}={\left[\begin{array}{ccc} u_{b1} & u_{b2} & \begin{array}{cc} \ldots & u_{b6} \end{array} \end{array}\right]}^{T}$, $\omega_{i}\left(i=1,2,\ldots ,6\right)$ and $\xi_{i}\left(i=1,2,\ldots ,6\right)$ are substituted into Equation (15) to obtain the load response u in modal coordinate by solving the ordinary differential equations. Finally, load response x is obtained by substituting u and S into Equation (13).

### 4.2. Theoretical Displacement Transmissibility

Considering the dynamic coupling of the 6-DOF bumper, base vibration excitation in one direction would cause displacement responses of load in six directions. Meanwhile, base vibration excitation could be from six directions, so displacement transmissibility of the bumper in six directions under six directions base vibration excitation are calculated, respectively, based on the calculation flowchart shown in Figure 5. The six directions include x-direction linear displacement, y-direction linear displacement, z-direction linear displacement, x-direction angular displacement, y-direction angular displacement and z-direction angular displacement.

For x-direction linear displacement, the base vibration excitation is defined as follows.

(16) $$x_{b}={\left[\begin{array}{cccccc} X_{b}\cos\left(\omega t\right) & 0 & 0 & 0 & 0 & 0 \end{array}\right]}^{T},$$

where $X_{b}$ is defined as the vibration amplitude, and ω is defined as the vibration frequency.

According to Equation (13), the base vibration excitation in modal coordinate is calculated as follows.

(17) $$u_{b}=S^{-1}x_{b}=\left[\begin{array}{c} u_{1}\\ \vdots \\ u_{6} \end{array}\right]=\left[\begin{array}{c} {\left(S^{-1}\right)}_{1,1}X_{b}\cos\left(\omega t\right)\\ \vdots \\ {\left(S^{-1}\right)}_{6,1}X_{b}\cos\left(\omega t\right) \end{array}\right],$$

where ${\left(S^{-1}\right)}_{i,j}$ means element intersecting in i th row and j th column of $S^{-1}$.

According to Equation (15), Equation (17) can be written as follows.

(18) $$\begin{array}{l} {\ddot{u}}_{i}\left(t\right)+2\xi_{i}\omega_{i}{\dot{u}}_{i}\left(t\right)+\omega_{i}^{2}u_{i}\left(t\right)=\omega_{i}^{2}{\left(S^{-1}\right)}_{i,1}X_{b}\cos\left(\omega t\right)-2\xi_{i}\omega_{i}\omega {\left(S^{-1}\right)}_{i,1}X_{b}\sin\left(\omega t\right),\\ \left(i=1,2,\ldots ,6\right) \end{array},$$

The solution of Equation (18) is set as follows.

(19) $$u_{i}=U_{i}\cos\left(\omega t-\varphi_{i}\right),$$

where $U_{i}$ is set as the response amplitude, and $\varphi_{i}$ is defined as the response phase angle.

Equation (19) is substituted into Equation (18); then Equation (18) can be written as follows.

(20) $$\begin{array}{l} U_{i}\left\{\left(\omega_{i}^{2}-\omega^{2}\right)\cos\varphi_{i}+2\xi_{i}\omega_{i}\omega \sin\varphi_{i}\right\}\cos\left(\omega t\right)+\\ U_{i}\left\{\left(\omega_{i}^{2}-\omega^{2}\right)\sin\varphi_{i}-2\xi_{i}\omega_{i}\omega \cos\varphi_{i}\right\}\sin\left(\omega t\right)\\ =\omega_{i}^{2}{\left(S^{-1}\right)}_{i,1}X_{b}\cos\left(\omega t\right)-2\xi_{i}\omega_{i}\omega {\left(S^{-1}\right)}_{i,1}X_{b}\sin\left(\omega t\right).\\ \left(i=1,2,\ldots ,6\right) \end{array},$$

Because Equation (20) can be established for arbitrary time t, the coefficients of $\cos\left(\omega t\right)$ and $\sin\left(\omega t\right)$ in the left of Equation (20) should be equal to that of Equation (20), respectively, as follows.

(21) $$\left\{\begin{array}{c} \begin{array}{l} U_{i}\left\{\left(\omega_{i}^{2}-\omega^{2}\right)\cos\varphi_{i}+2\xi_{i}\omega_{i}\omega \sin\varphi_{i}\right\}\\ =\omega_{i}^{2}{\left(S^{-1}\right)}_{i,1}X_{b}; \end{array}\\ \begin{array}{l} U_{i}\left\{\left(\omega_{i}^{2}-\omega^{2}\right)\sin\varphi_{i}-2\xi_{i}\omega_{i}\omega \cos\varphi_{i}\right\}\\ =-2\xi_{i}\omega_{i}\omega {\left(S^{-1}\right)}_{i,1}X_{b}. \end{array} \end{array}\right.,$$

By solving Equation (21), we can obtain the expressions of $U_{i}$ and $\varphi_{i}$ as follows.

(22) $$U_{i}=\frac{\sqrt{\omega_{i}^{4}+4\xi_{i}^{2}\omega_{i}^{2}\omega^{2}}}{\sqrt{{\left(\omega_{i}^{2}-\omega^{2}\right)}^{2}+4\xi_{i}^{2}\omega_{i}^{2}\omega^{2}}}{\left(S^{-1}\right)}_{i,1}X_{b},$$

(23) $$\varphi_{i}=act\tan\frac{2\xi_{i}\omega^{3}}{\omega_{i}\left\{4\xi_{i}^{2}\omega^{2}+\left(\omega_{i}^{2}-\omega^{2}\right)\right\}},$$

The theoretical response of the load in modal coordinate under x-direction linear displacement is obtained as presented in Equations (19), (22) and (23).

Finally, according to Equation (13), the theoretical response of the load in physical coordinate under x-direction linear displacement can be written as follows.

(24) $$x=\left[\begin{array}{c} x_{1}\\ \vdots \\ x_{6} \end{array}\right]=\left[\begin{array}{c} \sum_{j=1}^{6}{\left(S\right)}_{1,j}u_{j}\\ \vdots \\ \sum_{j=1}^{6}{\left(S\right)}_{6,j}u_{j} \end{array}\right],$$

where ${\left(S\right)}_{i,j}$ means element intersecting in i th row and j th column of S.

Similarly, responses of load under base vibration excitation in other five directions can be calculated.

The displacement transmissibility of the bumper is defined as the root-mean-square ratio of the response to that of the base vibration excitation which is illustrated in [34,35] as follows.

(25) $$T=\frac{RMS\left(x\right)}{RMS\left(x_{b}\right)},$$

where $RMS\left(•\right)$ represents the root- mean-square ratio of the displacement at the steady state; T represents the displacement transmissibility. For base vibration excitation in each direction, T is a vector containing six elements which represent transmissibility of response in six directions.

**Figure 6:**
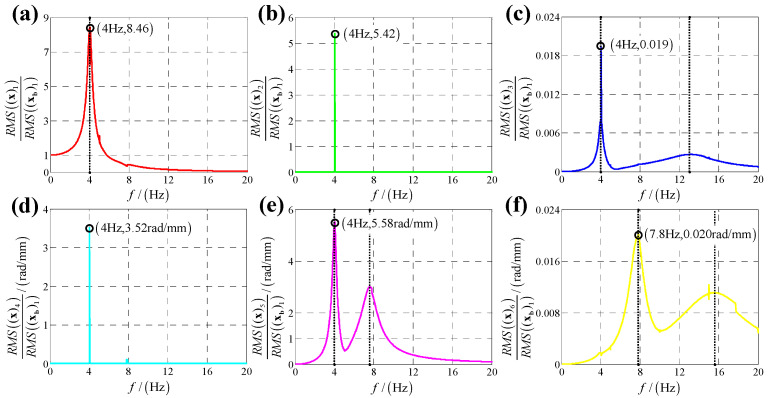
Displacement transmissibility under x-direction linear displacement base vibration excitation for (**a**) x-direction linear displacement response; (**b**) y-direction linear displacement response; (**c**) z-direction linear displacement response; (**d**) x-direction angular displacement response; (**e**) y-direction angular displacement response and (**f**) z-direction angular displacement response.

**Figure 7:**
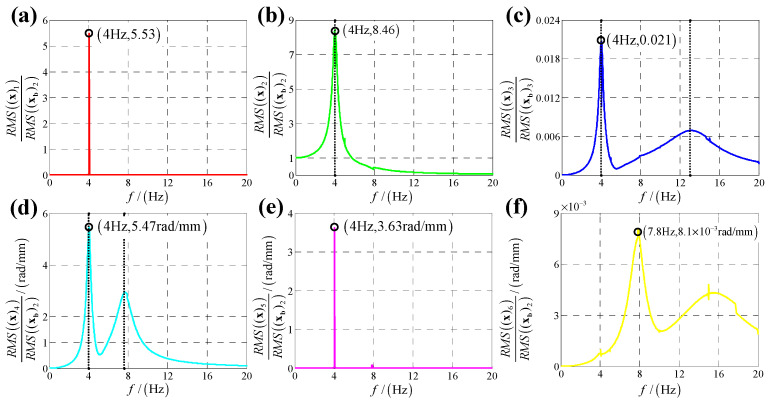
Displacement transmissibility under y-direction linear displacement base vibration excitation for (**a**) x-direction linear displacement response; (**b**) y-direction linear displacement response; (**c**) z-direction linear displacement response; (**d**) x-direction angular displacement response; (**e**) y-direction angular displacement response and (**f**) z-direction angular displacement response.

**Figure 8:**
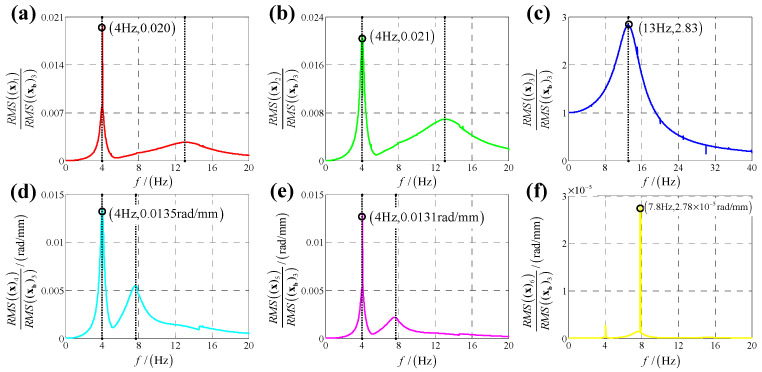
Displacement transmissibility under z-direction linear displacement base vibration excitation for (**a**) x-direction linear displacement response; (**b**) y-direction linear displacement response; (**c**) z-direction linear displacement response; (**d**) x-direction angular displacement response; (**e**) y-direction angular displacement response and (**f**) z-direction angular displacement response.

**Figure 9:**
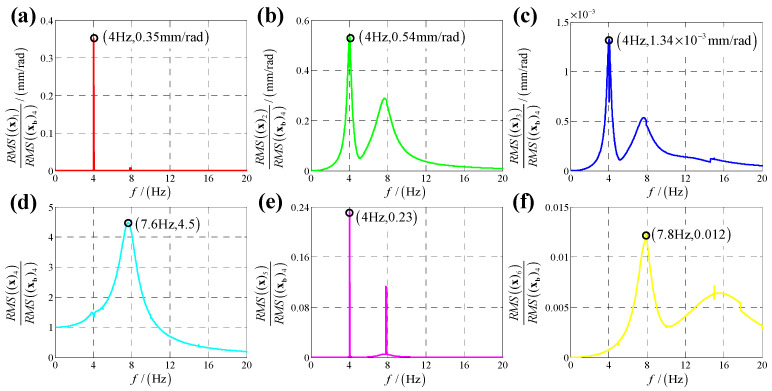
Displacement transmissibility under x-direction angular displacement base vibration excitation for (**a**) x-direction linear displacement response; (**b**) y-direction linear displacement response; (**c**) z-direction linear displacement response; (**d**) x-direction angular displacement response; (**e**) y-direction angular displacement response and (**f**) z-direction angular displacement response.

**Figure 10:**
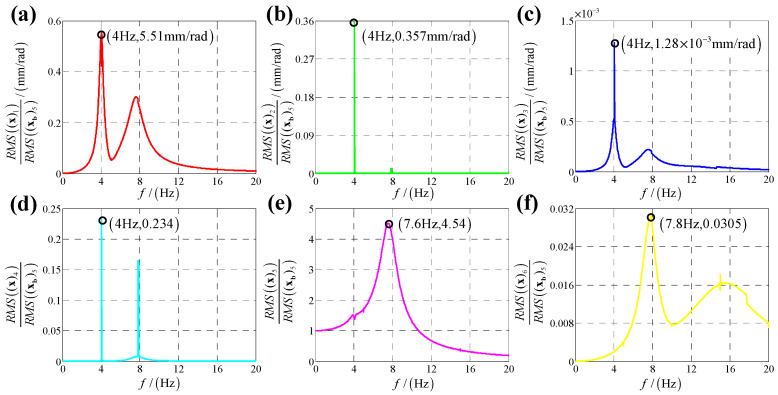
Displacement transmissibility under y-direction angular displacement base vibration excitation for (**a**) x-direction linear displacement response; (**b**) y-direction linear displacement response; (**c**) z-direction linear displacement response; (**d**) x-direction angular displacement response; (**e**) y-direction angular displacement response and (**f**) z-direction angular displacement response.

**Figure 11:**
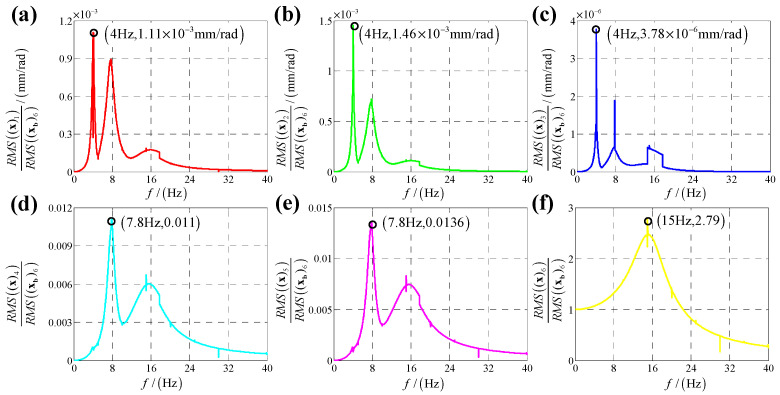
Displacement transmissibility under z-direction angular displacement base vibration excitation for (**a**) x-direction linear displacement response; (**b**) y-direction linear displacement response; (**c**) z-direction linear displacement response; (**d**) x-direction angular displacement response; (**e**) y-direction angular displacement response and (**f**) z-direction angular displacement response.

According to the calculation results of response, structural parameters in Table 1, dynamic parameters in Appendix A.6 and Equation (25), displacement transmissibility under six directions base vibration excitation are presented in Figure 6, Figure 7, Figure 8, Figure 9, Figure 10 and Figure 11.

Comparing Figure 6 with Figure 7 as well as Figure 9 with Figure 10, because the bumper is a symmetric structure around axis OZ, displacement transmissibility of x-direction and y-direction responses under x-direction linear (angular) displacement base vibration excitation has a mirror-image relationship with that under y-direction linear (angular) base vibration, and displacement transmissibility of z-direction response under x-direction linear (angular) displacement base vibration excitation is equal to that under y-direction linear (angular) base vibration.

For displacement transmissibility under x-direction linear displacement base vibration excitation, as shown in Figure 6, the x-direction linear response is like a linear single DOF system which only has a resonance frequency. However, due to dynamic coupling, x-direction linear displacement base vibration causes other five directions responses: y-direction linear response is equal to zero for all vibration frequencies except resonance frequency (in resonance frequency, displacement transmissibility for y-direction linear response has a significant sharp point); x-direction angular response is similar to y-direction linear response which has a non-ignorable sharp point in resonance frequency; y-direction angular response is like a nonlinear single DOF system which has two resonance frequencies and response of z-direction linear (angular) is small which can be regarded as zero meaning that x-direction linear vibration has almost no coupling effect on z-direct response.

For displacement transmissibility under y-direction linear displacement base vibration excitation, as shown in Figure 7, the x-direction and y-direction responses are mirrored with that under x-direction linear displacement vibration, and the z-direction response is equal to that under x-direction linear displacement vibration.

For displacement transmissibility under x-direction angular displacement base vibration excitation, as shown in Figure 9, x-direction angular response likes a linear single DOF system which only has a resonance frequency; responses in other five directions small enough to be ignored, so it can be concluded that x-direction angular vibration has little coupling effect on other five directions.

For displacement transmissibility under y-direction angular displacement base vibration excitation, as shown in Figure 10, the x-direction and y-direction responses are mirrored with that under x-direction angular displacement vibration, and the z-direction response is equal to that under x-direction angular displacement vibration.

For displacement transmissibility under z-direction linear (angular) displacement base vibration excitation, as shown in Figure 8 (Figure 11), z-direction linear (angular) response is like a linear single DOF system which only has a resonance frequency; responses in other five directions are small enough to be ignored, so it can be concluded that z-direction linear (angular) vibration has little coupling effect on other five directions.

In summary, it can be concluded that x-direction and y-direction linear displacement has a significant coupling effect on y-direction and x-direction responses, respectively, but has almost no coupling effect on z-direct response, and x-direction and y-direction angular vibration as well as both z-direction linear and angular vibration has little coupling effect on other five directions which can be ignored. The result can be explained by the stiffness metric obtained in terms of the geometry of the Stewart platform and the properties of the buffer as shown in Equation (26) (the small values are neglected). From Equation (26), the coupling relationships of six directions for the bumper agree with the theoretical results.

(26) $$K=\left[\begin{array}{ccc} \begin{array}{cc} k_{11} & 0\\ 0 & k_{22} \end{array} & \begin{array}{cc} 0 & 0\\ 0 & k_{24} \end{array} & \begin{array}{cc} k_{15} & 0\\ 0 & 0 \end{array}\\ \begin{array}{cc} 0 & 0\\ 0 & k_{24} \end{array} & \begin{array}{cc} k_{33} & 0\\ 0 & k_{44} \end{array} & \begin{array}{cc} 0 & 0\\ 0 & 0 \end{array}\\ \begin{array}{cc} k_{15} & 0\\ 0 & 0 \end{array} & \begin{array}{cc} 0 & 0\\ 0 & 0 \end{array} & \begin{array}{cc} k_{55} & 0\\ 0 & k_{66} \end{array} \end{array}\right],$$

## 5. Experimental Verification

In order to prove the validity of the proposed method, vibration experiments for the bumper are conducted to obtain the actual displacement transmissibility of the bumper under base vibration excitation. In practice, the bumper suffers from linear displacement base vibration excitation more commonly than angular displacement vibration, and the angular displacement is difficult to measure, but the linear displacement can be easily measured by displacement sensor. Therefore, vibration experiments only consider the linear displacement.

**Figure 12:**
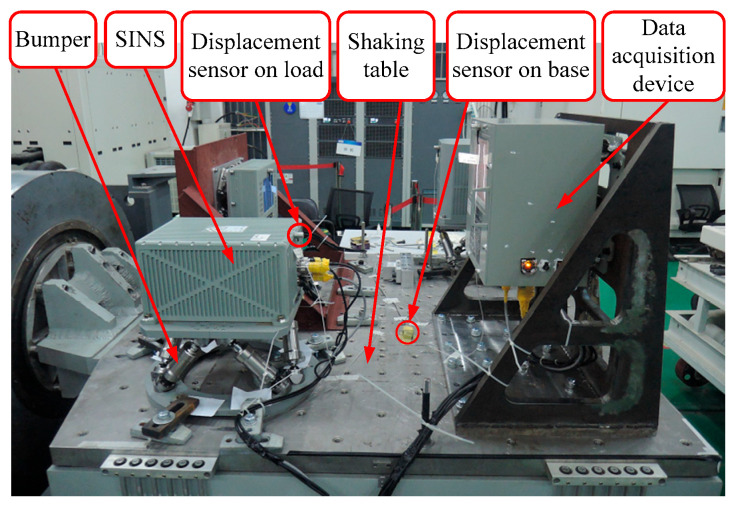
Vibration experiments setup for the bumper.

The experimental setup is shown in Figure 12, the SINS is fixed on the mobile base of the bumper and the bumper is mounted on the base of the shaking table. Two displacement sensors are fixed on the SINS and base of the shaking table to measure the linear displacement of the SINS and base, respectively. Finally, the data acquisition device gathers the data and calculates the displacement transmissibility defined as the root-mean-square ratio of the response to that of the base vibration excitation. The displacement sensor is CYQ-9250 (Tianjin Qixing Huakong Automation Instrument Co., Ltd., Tianjin, China). The shaking table is a customized electric vibration testing system (Suzhou Suling Environmental Testing Equipment Co., Ltd., Suzhou, China). The data acquisition device is a customized data collection box (Fujian Xinghai Communication Technology Co., Ltd., Fuzhou, China).

**Figure 13:**
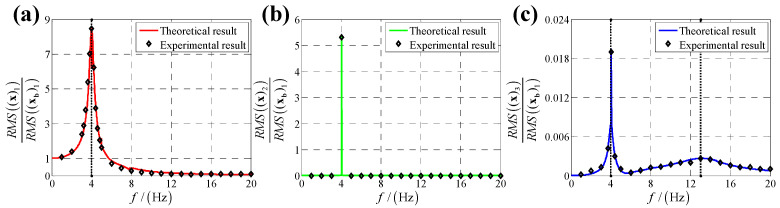
Experimental results and theoretical results of displacement transmissibility under x-direction linear displacement base vibration excitation for (**a**) x-direction linear displacement response; (**b**) y-direction linear displacement response and (**c**) z-direction linear displacement response.

**Figure 14:**
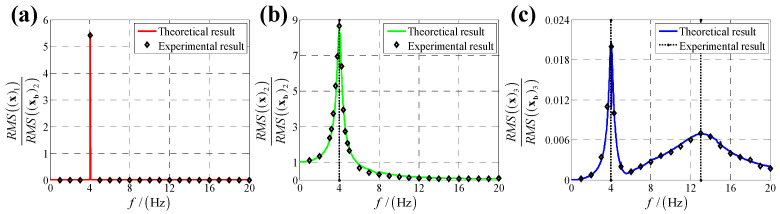
Experimental results and theoretical results of displacement transmissibility under y-direction linear displacement base vibration excitation for (**a**) x-direction linear displacement response; (**b**) y-direction linear displacement response and (**c**) z-direction linear displacement response.

**Figure 15:**
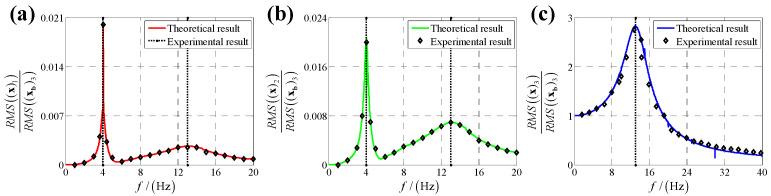
Experimental results and theoretical results of displacement transmissibility under z-direction linear displacement base vibration excitation for (**a**) x-direction linear displacement response; (**b**) y-direction linear displacement response and (**c**) z-direction linear displacement response.

For x-direction linear displacement base vibration excitation, the shaking table is controlled to drive base to move in x-direction as the displacement ${\left(x_{b}\right)}_{1}=\cos\left(\omega t\right)$ at an appointed ω, and displacement sensors on load and shaking table measure the real-time displacement of load and shaking table, respectively; then the data acquisition device gathers the data and calculates the displacement transmissibility based on Equation (25) for the specified ω. Next, we change ω and obtain displacement transmissibility for each ω. Finally, a vibration frequency-displacement transmissibility scatter diagram for x-direction linear displacement base vibration excitation is plotted based on the experimental data shown in Figure 13. Similarly, vibration frequency-displacement transmissibility scatter diagrams for y-direction and z-direction linear displacement base vibration excitation are presented in Figure 14 and Figure 15, respectively.

In Figure 13, Figure 14 and Figure 15, frequency-displacement transmissibility scatter diagrams based on experimental results match frequency-displacement transmissibility curve plotted using theoretical results well, and the maximum quantitative gap between theoretical and experimental results is 3.6%, verifying the proposed method.

A comparison of the effectiveness of the proposed method with other existing methods is listed in Table 2. The maximum quantitative gap between theoretical and experimental results is 0.88% lower than the other existing methods. Thus, the proposed method can give a high accuracy displacement transmissibility evaluation for Stewart platform based SINS’s bumper under base vibration excitation.

## 6. Conclusions

This work presents a displacement transmissibility analysis method for the Stewart platform based SINS’s bumper under base vibration excitation, fully considering the structural complexity and dynamic coupling of the 6-DOF bumper. In a dynamic model, the bumper is established as a lumped parameter model by defining dynamic matrices that includes stiffness matrix, damping matrix and mass matrix. For the calculation of theoretical displacement results, coupled dynamic equations of the 6-DOF system under base vibration excitation are derived based on the proposed lumped parameter model firstly; then the coupled dynamic equations are transferred to decoupled dynamic equations by decoupling method. Next, a calculation flowchart of the vibration isolation performance of the bumper is proposed based on the deduced decoupled equations firstly; theoretical results are obtained by the calculation flowchart. The theoretical results are finally verified by vibration experiments as the maximum quantitative gap between theoretical and experimental results is 3.6%.

Based on this analysis, it can be concluded that x-direction and y-direction linear displacement has a significant coupling effect on y-direction and x-direction responses, respectively, but has almost no coupling effect on z-direct response. Moreover, x-direction and y-direction angular vibration as well as both z-direction linear and angular vibration has little coupling effect on other five directions, which can be ignored.

This work also has some limitations that need to be solved in future work, which are as follows.

(1) Some simplifications (considering Jacobian matrix as a constant matrix, neglecting kinetic energy of buffer bars, ideal joints assumption) might limit the applicability of the results in more complex real-world scenarios.
(2) This work lacks sensitivity analysis to determine how structural parameter variations affect the displacement transmissibility.
(3) Nonlinear damping or frequency-dependent damping characteristics is not considered.

This work gives a detailed analytical approach for the displacement transmissibility of the 6-DOF bumper. It can contribute to the evaluation of vibration isolation performance for the bumper and other widely used Stewart platform based isolators. It can also contribute to further research in optimizing the buffer bars. In the future work, a more refined model will be established considering the changes of Jacobian matrix, kinetic energy of buffer bars, influences of joints and nonlinear damping, and sensitivity analyses will be conducted to determine the influence of structural parameters.

## Figures and Tables

**Table 1:** Structural parameters of the bumper.

h/mm	$R_{b}$/mm	$R_{a}$/mm	$\beta_{s}$/°	$\alpha_{m}$/°
174.02	250.00	250.00	14.58	14.58

**Table 2:** Comparison of the effectiveness of the proposed method with other existing methods.

Study	This Study	Zhang et al. [5]	Chen et al. [36]	Li et al. [37]
Maximum quantitative gap between theoretical and experimental results	3.60%	4.48%	5.32%	5.15%

**Figure A1:**
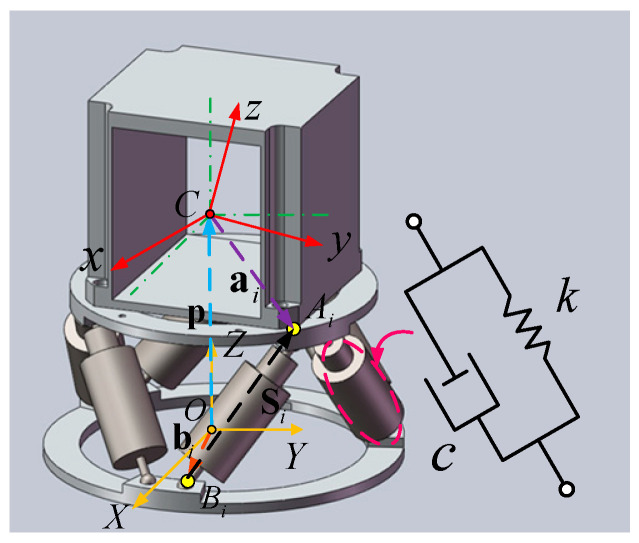
The coordinate definition and model of the bumper.

## Data Availability

Data will be made available upon request.

## Abbreviations

The following abbreviations are used in this manuscript:

SINS	Strap-down inertial navigation system
DOF	Degrees of freedom
h	Height of bumper
$R_{b}$	Radius of static platform
$R_{a}$	Radius of mobile platform
$\beta_{s}$	Half flare angle of adjoining spherical hinge on static platform
$\alpha_{m}$	Half flare angle of adjoining spherical hinge on mobile platform
O	Center of mass of static platform
O−XYZ	Static platform frame
C	Center of mass of load
C−xyz	Load frame
$p={\left[\begin{array}{ccc} x_{p} & y_{p} & z_{p} \end{array}\right]}^{T}$	Position vector of C in O−XYZ
$\Phi ={\left[\begin{array}{ccc} \alpha & \beta & \gamma \end{array}\right]}^{T}$	Euler-angles of mobile platform related to O−XYZ
x	Generalized coordinate of mobile platform in physical coordinate
$x_{b}$	Generalized coordinate of static platform in physical coordinate
M	Mass matrix of bumper
K	Stiffness matrix of bumper
C	Damping matrix of bumper
k	Stiffness of buffer bar
c	Damping of buffer bar
λ	Ratio of damping to stiffness for buffer bars
u	Generalized coordinate of mobile platform in modal coordinate
$u_{b}$	Generalized coordinate of static platform in modal coordinate
S	Coordinate transformation matrix between physical and modal coordinate
$\omega_{i}\left(i=1,2,\ldots ,6\right)$	Modal frequency
$\xi_{i}\left(i=1,2,\ldots ,6\right)$	Modal damping ratio
$X_{b}$	Vibration amplitude
ω	Vibration frequency
$U_{i}\left(i=1,2,\ldots ,6\right)$	Response amplitude in modal coordinate
$\varphi_{i}\left(i=1,2,\ldots ,6\right)$	Response phase angle in modal coordinate
T	Displacement transmissibility

## Appendix A

### Appendix A.1

Terminology table for Appendix A is Table A1.

**Table A1:** Terminology table for Appendix A.

Symbol	Parameter Definition
O	Center of mass of static platform
O−XYZ	Static platform frame
C	Center of mass of load
C−xyz	Load frame
p	Position vector of C in O−XYZ
$A_{i}$	Upper spherical hinges centers
$a_{i}$	$\mathrm{Coordinates}\;\mathrm{of}\;A_{i}$ in C−xyz
$B_{i}$	Lower spherical hinges centers
$b_{i}$	$\mathrm{Coordinates}\;\mathrm{of}\;B_{i}$ in O−XYZ
$S_{i}$	Vectors of buffer bars in O−XYZ
Φ	Euler-angles of mobile platform related to O−XYZ
x	Generalized coordinate of mobile platform
R	Rotation matrix of platform
ω	Rotation angular velocity of mobile platform
$s_{i}$	Unit vectors of buffer bars in O−XYZ
$l_{i}$	Length scalars of buffer bars
k	Stiffness of buffer bar
c	Damping of buffer bar
m	Mass of load
$I_{C}$	Inertial tensor with respect to point C in frame C−xyz

### Appendix A.2

Coordinate definition and model for the bumper are illustrated in Figure A1. Point O is the center of mass of the static platform, and the static platform frame O−XYZ is fixed on the static platform. As the weight of buffer bars is negligible related to the weight of the SINS and mobile platform, the weight of buffer bars is ignored, and the load of bumper is regarded as the assembly of SINS and mobile platform. Point C is the center of mass of the load, and the load frame C−xyz is bound on the load.

Vector $p={\left[\begin{array}{ccc} x_{p} & y_{p} & z_{p} \end{array}\right]}^{T}$ denotes the position vector of point C in O−XYZ; points $A_{i}(i=1,2,\ldots ,6)$ denote the center of the upper spherical hinges, and vectors $a_{i}(i=1,2,\ldots ,6)$ denote the coordinates of the $A_{i}(i=1,2,\ldots ,6)$ in C−xyz; points $B_{i}(i=1,2,\ldots ,6)$ denote the center of the lower spherical hinges, and vectors $b_{i}(i=1,2,\ldots ,6)$ denote the coordinates of the $B_{i}(i=1,2,\ldots ,6)$ in O−XYZ; vectors $S_{i}(i=1,2,\ldots ,6)$ denote the vectors of each buffer bar, which is from $B_{i}$ to $A_{i}$ in O−XYZ. The attitude of mobile platform related to static platform is defined as Euler angles $\Phi ={\left[\begin{array}{ccc} \alpha & \beta & \gamma \end{array}\right]}^{T}$, in which mobile platform firstly rotates α around axis OX, then rotates β around axis OY and finally rotates γ around axis OZ. Moreover, vector x is set as the generalized coordinate of the mobile platform and can be written as follows.

(A1) $$x={\left[\begin{array}{cc} p & \Phi \end{array}\right]}^{T}={\left[x_{p},y_{p},z_{p},\alpha ,\beta ,\gamma \right]}^{T},$$

### Appendix A.3

Jacobian matrix is concisely descriptive of the relationship between the mobile platform generalized velocity and the buffer bars’ velocities.

Vector $S_{i}$ can be expressed as follows.

(A2) $$S_{i}=p+Ra_{i}-b_{i},$$

where matrix R represents the rotation matrix of the frame C−xyz related to the frame O−XYZ; as the Euler angle is small, matrix R can be written as follows.

(A3) $$R=\left[\begin{array}{ccc} 1 & \gamma & -\beta \\ -\gamma & 1 & \alpha \\ \beta & -\alpha & 1 \end{array}\right],$$

Both sides of Equation (A2) are differentiated by time as follows.

(A4) $${\dot{S}}_{i}=\dot{p}+\omega \times Ra_{i},$$

where ω is the rotation angular velocity vector of mobile platform related to static platform.

$s_{i}(i=1,2,\ldots ,6)$ is defined as the unit vector of each buffer bar, and $l_{i}(i=1,2,\ldots ,6)$ is set as the length scalar of each buffer bar. The differential of $l_{i}$ related to time can be rewritten as follows.

(A5) $${\dot{l}}_{i}=s_{i}^{T}\cdot {\dot{S}}_{i},$$

Equation (A4) is substituted into Equation (A5), and then Equation (A5) can be rewritten as follows.

(A6) $${\dot{l}}_{i}=s_{i}^{T}\cdot \dot{p}+s_{i}^{T}\cdot (\omega \times Ra_{i}),$$

According to the computational formula for the mixed product of three vectors, Equation (A6) can be transformed as follows.

(A7) $${\dot{l}}_{i}=s_{i}^{T}\cdot \dot{p}+{\left(c_{i}\times s_{i}\right)}^{T}\cdot \omega ,$$

where $c_{i}=Ra_{i}$. When i=1,2,…,6, Equation (A7) can be rewritten to matrix form as follows.

(A8) $$\dot{l}=J_{x}\cdot \dot{x},$$

where l is a vector constructed by all six length scalars of buffer bars, and $J_{x}$ is the Jacobian matrix from 6-DOF movement of mobile platform to bidirectional linear displacements of buffer bars, and l and $J_{x}$ can be expressed as follows.

(A9) $$l={\left[\begin{array}{cccccc} l_{1} & l_{2} & l_{3} & l_{4} & l_{5} & l_{6} \end{array}\right]}^{T},$$

(A10) $$J_{x}={\left[\begin{array}{cc} \begin{array}{c} s_{1}^{T}\\ s_{2}^{T} \end{array} & \begin{array}{c} {\left(c_{1}\times s_{1}\right)}^{T}\\ {\left(c_{2}\times s_{2}\right)}^{T} \end{array}\\ \begin{array}{c} \vdots \\ s_{6}^{T} \end{array} & \begin{array}{c} \vdots \\ {\left(c_{6}\times s_{6}\right)}^{T} \end{array} \end{array}\right]}_{6\times 6},$$

### Appendix A.4

As shown in Figure A1, each buffer bar is modeled as a spring-damper consisting of a spring and a damper in parallel, and the stiffness and damping of the buffer bar’s model are k and c, respectively. $F_{k}$ and $F_{c}$ are defined as the generalized forces on load due to elastic forces $f_{k}$ and damping forces $f_{c}$ of buffer bars, respectively. $f_{k}$ and $f_{c}$ can be expressed as follows.

(A11) $$f_{k}=k\cdot \Delta l=k\cdot {\left[\begin{array}{cccccc} \Delta l_{1} & \Delta l_{2} & \Delta l_{3} & \Delta l_{4} & \Delta l_{5} & \Delta l_{6} \end{array}\right]}^{T},$$

(A12) $$f_{c}=c\cdot \dot{l}=c\cdot {\left[\begin{array}{cccccc} {\dot{l}}_{1} & {\dot{l}}_{2} & {\dot{l}}_{3} & {\dot{l}}_{4} & {\dot{l}}_{5} & {\dot{l}}_{6} \end{array}\right]}^{T},$$

Based on the principle of virtual work, the work of $F_{k}$ on tiny change of x is equal to the work of $f_{k}$ on small variation of l as follows.

(A13) $$F_{k}^{T}\cdot \Delta x=f_{k}^{T}\cdot \Delta l,$$

Equations (A8) and (A11) are substituted into Equation (A13). Equation (A13) can be written as follows.

(A14) $$F_{k}=K\cdot \Delta x=(kJ_{x}^{T}J_{x})\cdot \Delta x,$$

where K is the stiffness matrix of the bumper and can be expressed as:

(A15) $$K=kJ_{x}^{T}J_{x},$$

Similarly, the damping matrix of the bumper C can be expressed as:

(A16) $$C=cJ_{x}^{T}J_{x},$$

### Appendix A.5

The mass matrix of the bumper with respect to point O in frame O−XYZ can be written as follows.

(A17) $$M=\left[\begin{array}{cc} mI_{3\times 3} & 0_{3\times 3}\\ 0_{3\times 3} & I_{O} \end{array}\right],$$

where m is the mass of the load; $I_{3\times 3}$ is 3×3 identity matrix; $0_{3\times 3}$ is 3×3 null matrix; $I_{O}$ is the inertial tensor with respect to point O in frame O−XYZ.

$I_{C}$ is the inertial tensor with respect to point C in frame C−xyz which is written as:

(A18) $$I_{C}=\left[\begin{array}{ccc} I_{xx} & I_{xy} & I_{xz}\\ I_{xy} & I_{yy} & I_{yz}\\ I_{xz} & I_{yz} & I_{zz} \end{array}\right],$$

According to the parallel axis theorem and rotation transformation of coordinate systems, $I_{O}$ can be expressed by $p={\left[\begin{array}{ccc} x_{p} & y_{p} & z_{p} \end{array}\right]}^{T}$, R and $I_{C}$ as follows.

(A19) $$I_{O}=R\left[\begin{array}{ccc} I_{xx}+m(y_{p}^{2}+z_{p}^{2}) & I_{xy}+mx_{p}y_{p} & I_{xz}+mx_{p}z_{p}\\ I_{xy}+mx_{p}y_{p} & I_{yy}+m(x_{p}^{2}+z_{p}^{2}) & I_{yz}+my_{p}z_{p}\\ I_{xz}+mx_{p}z_{p} & I_{yz}+my_{p}z_{p} & I_{zz}+m(x_{p}^{2}+y_{p}^{2}) \end{array}\right]R^{T},$$

### Appendix A.6

Dynamic parameters of the bumper are determined by 3D modeling software, and they are listed as follows.

**Table A2:** Dynamic parameters of the bumper.

Dynamic Parameters	Values
$p={\left[\begin{array}{ccc} x_{p} & y_{p} & z_{p} \end{array}\right]}^{T}$	$\left[\begin{array}{ccc} 0.308 & -0.794 & 299.404 \end{array}\right]$ mm
k	44,000 N/m
c	200 N·s/s
m	30.78 kg
$I_{C}$	$\left[\begin{array}{ccc} 0.2612 & 0.0002 & 0.0011\\ 0.0002 & 0.3210 & 0.0028\\ 0.0011 & 0.0028 & 0.2554 \end{array}\right]$ kg·m^2^
